# Structure and function of class III pistil-specific extensin-like protein in interspecific reproductive barriers

**DOI:** 10.1186/s12870-019-1728-8

**Published:** 2019-03-29

**Authors:** Camila M. L. Alves, Andrzej K. Noyszewski, Alan G. Smith

**Affiliations:** Department of Horticultural Science, University of Minnesota, Saint Paul, MN 55108 USA

**Keywords:** Gene complementation, 3’UTR RNAi, *Nicotiana tabacum*, *Nicotiana obtusifolia*, Arabinogalactan proteins, PELPIII, TTS, Pollen tube growth

## Abstract

**Background:**

The transmitting tissue of the style is the pathway for pollen tube growth to the ovules and has components that function in recognizing and discriminating appropriate pollen genotypes. In *Nicotiana tabacum*, the class III pistil extensin-like (PELPIII) arabinogalactan protein is essential for the inhibition of *N. obtusifolia* pollen tube growth. The transmitting tissue-specific (TTS) arabinogalactan protein amino acid sequence and expression pattern is similar to PELPIII, but it facilitates self-pollinated *N. tabacum*. The TTS and PELPIII arabinogalactan protein can be divided into the less conserved N-terminal (NTD) and the more conserved C-terminal (CTD) domains. This research tested whether the NTD is the key domain in determining PELPIII function in the inhibition of interspecific pollen tube growth. Three variant PELPIII gene constructs were produced where the PELPIII NTD was exchanged with the TTS NTD and a single amino acid change (cysteine to alanine) was introduced into the PELPIII NTD. The PELPIII variants of *N. tabacum* were tested for activity by measuring the inhibition *N. obtusifolia* pollen tube growth by using them to complement a 3’UTR RNAi transgenic line with reduced PELPIII mRNA.

**Results:**

The RNAi *N. tabacum* line had reduced PELPIII mRNA accumulation and reduced inhibition of *N. obtusifolia* pollen tube growth, but had no effect on self-pollen tube growth or pollen tube growth of 12 other *Nicotiana* species. The NTD of PELPIII with either the PELPIII or TTS CTDs complemented the loss PELPIII activity in the RNAi transgenic line as measured by inhibition of *N. obtusifolia* pollen tube growth. The TTS NTD with the PELPIII CTD and a variant PELPIII with a cysteine to alanine mutation in its NTD failed to complement the loss of PELPIII activity and did not inhibit *N. obtusifolia* pollen tube growth.

**Conclusion:**

The NTD is a key determinant in PELPIII’s function in regulating interspecific pollen tube growth and is a first step toward understanding the mechanism of how PELPIII NTD regulates pollen tube growth.

**Electronic supplementary material:**

The online version of this article (10.1186/s12870-019-1728-8) contains supplementary material, which is available to authorized users.

## Background

Interspecific reproductive barriers preserve species integrity [1], but for plant breeders the barriers are a hindrance to the introgression of genes from related species. In *Nicotiana*, the class III pistil-extensin like arabinogalactan protein (PELPIII; AGP) is essential for the reproductive barriers of *N. tabacum* pistils with *N. obtusifolia* and *N. repanda* pollen [2]*.* The PELPIII protein has amino acid sequence similarity to the transmitting tissue-specific (TTS) AGP that facilitates *N. tabacum* self-pollen tube growth [3, 4]. To better understand the relationship between PELPIII structure and its regulation of interspecific incompatibility and determine how two similar proteins have divergent functions, a domain swapping strategy between PELPIII and TTS proteins was developed.

*Nicotiana* is a model organism for research on pollen tube growth (PTG) because of the diversity of species, genetic and genomic resources, ease of transformation and large flowers. The *Nicotiana* transmitting tissue (TT) is the pathway for PTG from the stigma to the ovules and is where pre-zygotic interspecific reproductive barriers occur [5, 6]. The TT functions in PTG guidance, nutrition, and regulation of both self and interspecific PTG [7]. The mechanisms of how the TT interacts with pollen tubes or regulates PTG are not fully understood, but AGPs were shown to have a role in regulating PTG. The AGPs are known to regulate PTG as well as having diverse functions in vegetative growth, programmed cell death, molecular interactions, signaling, and development [8]. The *N. tabacum* PELPIII, TTS and 120 kDa (120 K) AGPs are specifically expressed in the TT and involved in PTG regulation. The AGPs are abundant in the *N. tabacum* TT and are secreted into the extracellular matrix [3, 9, 10].

The *N. tabacum* PELPIII is translocated from the TT extracellular matrix into the callose layer and callose plugs of *N. tabacum* and *N. obtusifolia* pollen tubes [6]. To determine whether PELPIII functions in regulating PTG [11] produced a PELPIII-antisense *N. tabacum* line with undetectable levels of PELPIII AGP and pollinated it with *N. tabacum, N. rustica* or *N. maritima* pollen. No differences in PTG between the antisense lines and normal plants occurred. However, these plants did have reduced PTG inhibition of *N. obtusifolia* and *N. repanda* [10]. Thus, PELPIII regulates PTG in a species-specific manner. PELPIII and TTS, are post-translationally modified through the hydroxylation of prolyl residues [8], as well as *O-*glycosylation [12, 13]. TTS was deglycosylated during PTG, which may provide energy in the form of carbohydrates for PTG [3, 4]. PELPIII was not deglycosylated during PTG and, consequently, may not provide carbohydrates to the pollen tubes [12], which may be a defining difference between PELPIII and TTS functions. The 120 K AGP has homology with PELPIII and is required for *N. alata S*-locus specific pollen rejection [14, 15]. However, the function of the 120 K in self-compatible *N. tabacum* is not known [9, 14, 15].

**Fig. 1:**
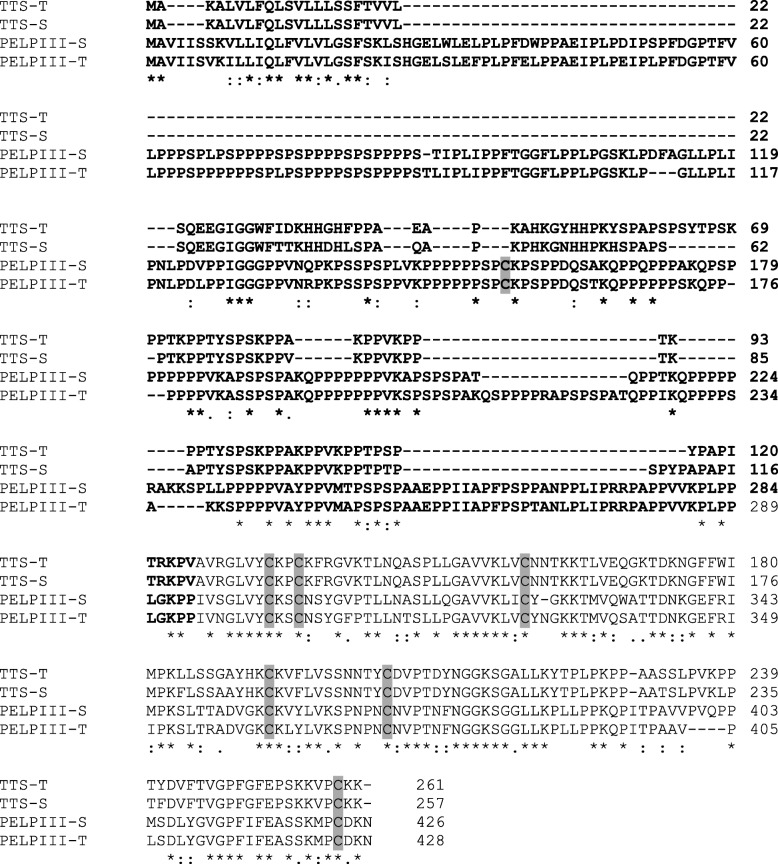
Comparison of the PELPIII-S and -T to TTS-S and -T. The PELPIII-S and -T (accession Z14019.1 and Z14015.1, respectively) amino acid sequences have 89.6% fully conserved residues and the TTS-S and -T (accession Z16403.1 and Z16404.1) amino acid sequences have 92.9% fully conserved residues. The PELPIII-S and TTS-S NTD amino acid sequences (bold) have 39.8% fully conserved and 58.7% similar residues. The PELPIII-S and TTS-S CTD amino acid sequences have 54.4% fully conserved and 75.2% similar residues. Highlighted in gray are the six cysteines in common between TTS and PELPIII and a unique cysteine in the PELPIII NTD. Asterisks represent fully conserved residues, colons represent amino acids with strong similar properties, and periods represent amino acids with weak similar properties

The PELPIII and TTS AGPs were divided into N-terminal (NTD) and C-terminal domains (CTD) [14, 16]. The PELPIII and TTS NTD is less conserved between AGPs and the CTD contains a highly conserved pattern of six cysteines [15, 17]. *Nicotiana tabacum* is an allotetraploid with two homeologous PELPIII and TTS genes from the ancestral parents *N. sylvestris* (PELPIII-S and TTS-S) and *N. tomentosiformis* (PELPIII-T and TTS-T). The PELPIII-S and T have 89.6% amino acid identity and TTS-S and T have 92.9% identity (Fig. 1). The high identity between PELPIII-S and -T and TTS-S and -T suggest that -S and -T have conserved functions. The PELPIII, TTS and 120 K CTDs are highly conserved among Nicotiana species each having two intrinsically disordered regions (IDR). One IDR is located in the NTD and the other IDR located in the CTD [15, 16]. Intrinsically disordered regions do not form regular secondary structures, are predicted to be highly glycosylated and may be a site of protein-protein interactions [15, 18–20]. The NTD amino acid sequences and predicted glycosylation patterns among *Nicotiana* species is polymorphic [15, 16]. The PELPIII NTD has a unique cysteine at position 156 that is not found in TTS or 120 K and may have an important role in protein structure and function. Cysteines form disulfide bonds and are important in protein folding and stability [21, 22]. The higher diversity, unique cysteine and potential for protein interactions led to the hypothesis that the NTD domain is essential for PELPIII’s activity in the inhibition of interspecific PTG.

**Fig. 2:**
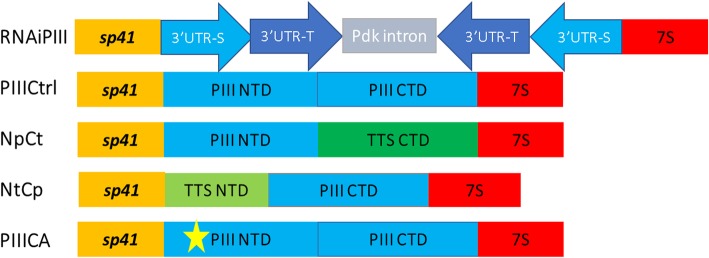
Gene constructs for expression of RNAi and PELPIII variants. Gene constructs used to test the hypothesis that the NTD domain is essential for PELPIII’s activity in the inhibition of interspecific PTG. All gene constructs had the *sp41* modified TT-specific promoter and the 7S: 3’UTR from the 7S seed storage protein of soybean. The RNAiPIII gene construct expresses both the 3’UTR-S and the 3’UTR-T. The RNAiPIII construct has the 3’UTRsequences in the sense and antisense with a Pdk intron between them.. The Pdk intron is the pyruvate dehydrogenase kinase intron [38]. The PIIICtrl gene construct has the PELPIII NTD followed by the PELPIII CTD. The NpCt gene construct has the PELPIII NTD followed by the TTS CTD. The NtCp gene construct has the TTS NTD followed by the PELPIII CTD. The PIIICA gene construct has the PELPIII NTD with the unique cysteine mutated to alanine at position 156 followed by the PELPIII CTD

Domain swapping and amino acid mutations are proven strategies to test the relationships between primary amino acid sequence and the function of a protein [23, 24]. PELPIII variants were produced by swapping the PELPIII and TTS domains and mutating the unique PELPIII NTD cysteine to alanine to test whether the NTD or CTD of the *N. tabacum* PELPIII is essential for the inhibition of *N. obtusifolia* PTG (Fig. 2). Transgenic *N. tabacum* lines with reduced levels of PELPIII mRNA were produced and subsequently crossed with lines expressing the variant PELPIII gene constructs to test their ability to complement the loss of PELPIII. The NTD from PELPIII combined with the CTD from PELPIII or TTS complemented the loss of normal PELPIII as measured by the inhibition of *N. obtusifolia* PTG. The PELPIII variants with the TTS NTD combined with the PELPIII CTD or the variant with NTD cysteine mutated to alanine did not complement the loss of normal PELPIII. Thus, the PELPIII NTD is necessary for *N. obtusifolia* PTG inhibition.

## Results

### NTD and CTD amino acid sequence analyses

**Fig. 3:**
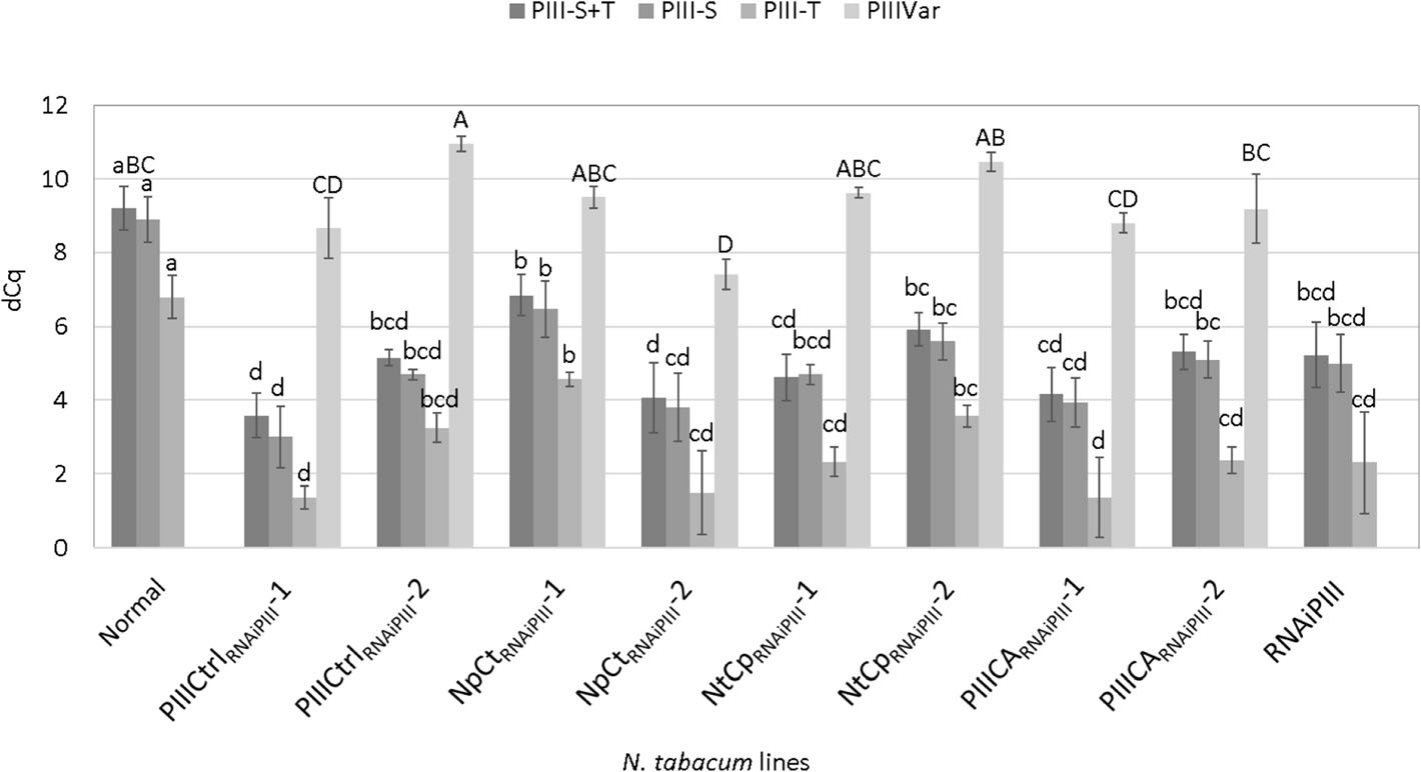
Accumulation of PELPIII -S, −T and variant transcripts in normal, complementation and RNAiPIII lines. The dCq is a log_2_ scale, calculated from two technical replicates and three biological replicates and actin was used as the reference gene. The fold change was calculated by dividing the mRNA levels being compared after converting from log_2_ to linear values. The error bars are standard deviations. Letters represent Tukey’s HSD mean separation at α = 0.05 among each PELPIII type (S, T, S + T and variant PELPIII) evaluated. Capital letters compares PELPIII-S + T mRNA accumulation in normal plants to PELPIII variant mRNA accumulation in other lines. Lower case letters compare S + T, S and T PELPIII mRNA accumulation among all *N. tabacum* lines. Normal: non-transgenic *N. tabacum* with wildtype levels of PELPIII (S and T); Complementation lines: PIIICtrl_RNAiPIII_ independent transformant lines 1 and 2 with the PIIICtrl and RNAiPIII transgenes; NpCt_RNAiPIII_ independent transformant lines 1 and 2 has the NpCt (PELPIII NTD and TTS CTD) and RNAiPIII transgenes; NtCp_RNAiPIII_ independent transformant lines 1 and 2 has the NtCp (TTS NTD and PELPIII CTD) and RNAiPIII transgenes; PIIICA_RNAiPIII_ independent transformant lines 1 and 2 has the PIIICA (cysteine to alanine mutation in PELPIII NTD) and RNAiPIII transgenes; RNAiPIII has the RNAiPIII transgene (reduced PELPIII-S and T) that was crossed with variant PELPIII transgenic lines to produce the complementation lines. PIIIVar is the mRNA accumulation from the control or complementation gene constructs

The two PELPIII genes have very similar amino acid sequences and are both transcriptionally active (Fig. 3) [16]. The level of PELPIII-S mRNA accumulation was higher than PELPIII-T in normal plants (Fig. 3). The alignment between homeologous TTS-S and -T, and PELPIII-S and -T showed 92.9 and 89.6% identity, respectively (Fig. 1) suggesting that TTS-S and -T, and PELPIII-S and -T have conserved activity. Figure 1 shows the significant similarity in amino acid sequence between the homeologous PELPIII-S and -T proteins that indicates their conserved function. Whereas the PELPIII and TTS proteins, regardless of S or T version, show much less similarity suggesting distinct functions. TTS and PELPIII NTDs have 121 and 289 amino acids, respectively, and TTS and PELPIII CTDs have 137 amino acids each (Fig. 1). The NTDs and CTDs of PELPIII-S and TTS-S have 39.8 and 54.4% fully conserved residues and 58.7 and 75.2% similarities, respectively (Fig. 1).

### Regulation of *Nicotiana* species pollen tube growth by *N. tabacum* PELPIII

The RNAiPIII transgenic plant selected for analysis had a 16-fold reduced PELPIII (−S plus -T) mRNA relative to styles from normal plants (Fig. 3). Pollination of normal and RNAiPIII plants was performed using pollen from 14 different *Nicotiana* species and two genotypes of *N. obtusifolia* to test if the reduction of PELPIII levels changed PTG compared to normal plants (Additional file 1). Pollen tubes from both *N. obtusifolia* and *N. obtusifolia* var. palmeri grew longer after 40 h in the RNAiPIII transgenic line relative to normal styles, indicating a loss of PELPIII-mediated inhibition in *N. tabacum* (Additional file 1). Pollination with 13 other *Nicotiana* species showed no significant differences in PTG in the RNAiPIII vs. normal styles. *Nicotiana stocktonii*, *N. suaveolens*, *N. veluntina* and *N. repanda* exhibited PTG inhibition soon after pollen germination, but were not different in RNAiPIII vs. normal styles (Additional file 1).

### Levels of PELPIII mRNA in complementation lines

Complementation lines, F1 heterozygous lines were produced by crossing the T0 heterozygous RNAiPIII line and a T0 heterozygous control (PIIICtrl) or variant transgenic line. Quantitative reverse transcription PCR (qRT-PCR) was used to measure mRNA levels using actin as a reference gene for normalization. All of the selected complementation lines had reduced endogenous PELPIII (S plus T) mRNA accumulation not statistically different to the levels measured in RNAiPIII (Fig. 3). The endogenous level of PELPIII in complementation lines would allow *N. obtusifolia* pollen to grow longer in the absence of complementation from a functional PELPIII variant construct. Variant PELPIII mRNA accumulation in PIIICtrl_RNAiPIII_-1, NpCt_RNAiPIII_-1, NtCp_RNAiPIII_-1 and 2 and PIIICA_RNAiPIII_-1 and 2 was not significantly different from endogenous PELPIII (S plus T) in normal plants. Whereas, the variant PELPIII mRNA accumulation in NpCt_RNAiPIII_-2 was 3.4-fold higher and the variant PELPIII mRNA accumulation in NpCt_RNAiPIII_-1 was 3.5-fold lower than the endogenous PELPIII (S plus T) mRNA accumulation in normal plants (Fig. 3).

### NTD of PELPIII is required for inhibition of *N. obtusifolia* PTG

**Fig. 4:**
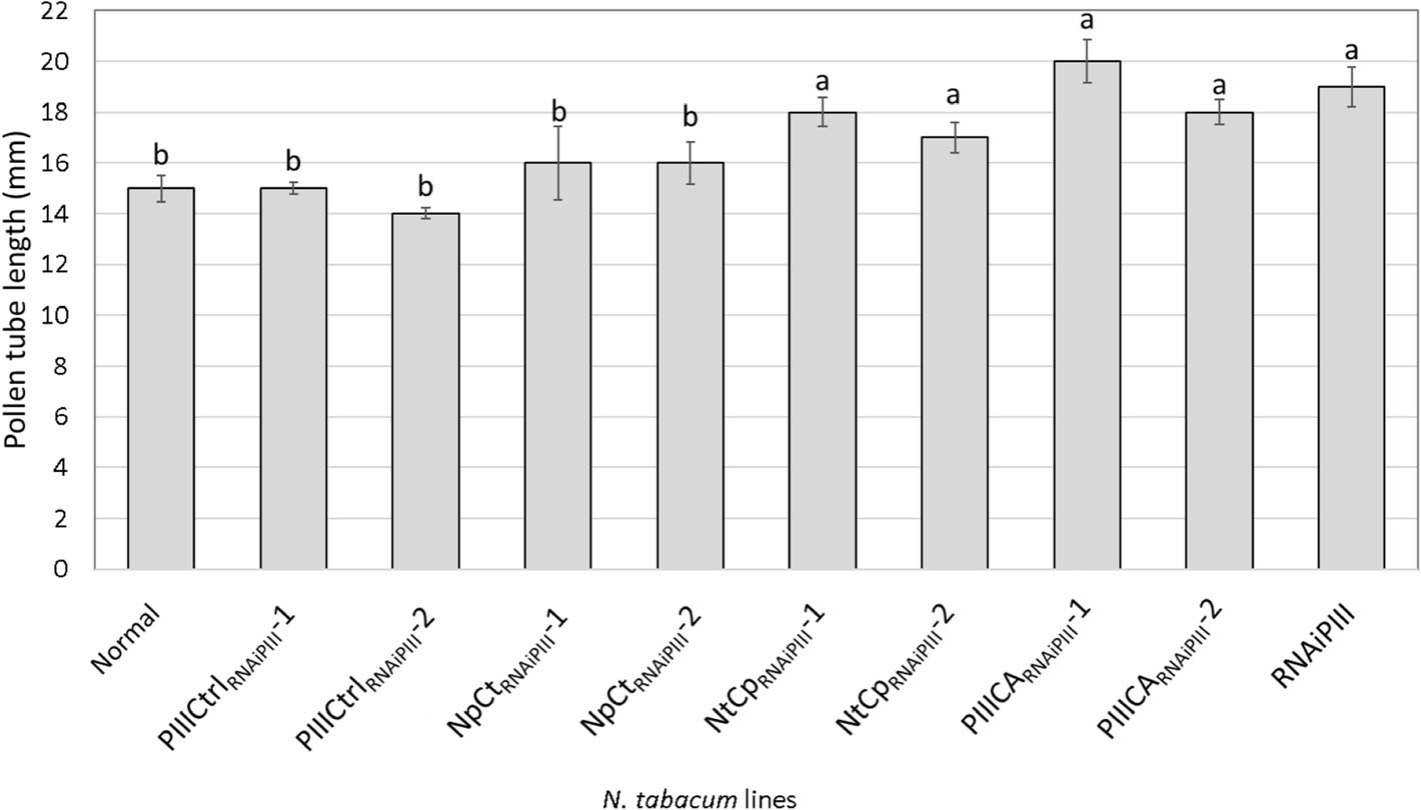
Mean *N. obtusifolia* PTG among normal, RNAiPIII and variant transgenic *N. tabacum* lines. Pollen tube length ± standard deviation was measured 40 h post pollination. The pollen tube length is the mean of five styles replicated three times. Different letters indicate a significant difference in PTG between a genotype and normal as determined by a Dunnett’s test at α = 0.05. Normal: non-transgenic *N. tabacum* with wildtype levels of endogenous PELPIII (S + T); Complementation lines: PIIICtrl_RNAiPIII_ independent transformant lines 1 and 2 with the PIIICtrl and RNAiPIII transgenes; NpCt_RNAiPIII_ independent transformant lines 1 and 2 has the NpCt (PELPIII NTD and TTS CTD) and RNAiPIII transgenes; NtCp_RNAiPIII_ independent transformant lines 1 and 2 has the NtCp (TTS NTD and PELPIII CTD) and RNAiPIII transgenes; PIIICA_RNAiPIII_ independent transformant lines 1 and 2 has the PIIICA (cysteine to alanine mutation in PELPIII NTD) and RNAiPIII transgenes; RNAiPIII has the RNAiPIII transgene (reduced PELPIII-S and T) that was crossed with variant PELPIII transgenic lines to produce the complementation lines

Pollinations of normal, RNAiPIII and transgenic complementation lines with *N. obtusifolia* pollen were used to measure the activity of complementing variant PELPIII gene construct (Fig. 4). The *N. obtusifolia* PTG was not significantly different among normal and PIIICtrl_RNAiPIII_-1 or − 2 styles, showing that the modified *sp41* promoter and 7S 3’UTR produced sufficient levels of PELPIII to complement the reduction of PELPIII in RNAiPIII styles, producing PTG similar to that in normal styles. *N. obtusifolia* PTG was not significantly different in PIIICtrl_RNAiPIII_-1, 2, NpCt_RNAiPIII_-1, 2 or normal styles (Fig. 4). The lower level of NpCt mRNA in NpCt_RNAiPIII_-2 compared to NpCt_RNAiPIII_-1 was still sufficiently high to significantly reduce the length of *N. obtusifolia* PTG. Therefore, the NpCt_RNAiPIII_ lines fully complemented the reduced activity of PELPIII due to the RNAiPIII (Fig. 4). NtCp_RNAiPIII_-1 and 2 had NtCp mRNA accumulation similar to endogenous PELPIII (S plus T) in normal plants and to control PELPIII mRNA accumulation in PIIICtrl_RNAiPIII_. However, *N. obtusifolia* PTG in the NtCp_RNAiPIII_-1 lines was significantly longer than in normal styles and the PIIICtrl_RNAiPIII_ styles (Figs. 4). Therefore, the NtCp gene construct failed to complement the reduction of PELPIII mRNA levels in the RNAiPIII transgenic lines. Similarly, the PIIICA construct did not complement the reduced levels of PELPIII mRNA levels, resulting in longer *N. obtusifolia* PTG relative to growth in normal styles (Fig. 4). Taken together, the mRNA accumulation and *N. obtusifolia* PTG results showed the PIIICtrl and NpCt constructs complemented reduction of PELPIII mRNA levels. The complementing variant PELPIII protein levels must be at a sufficient level and processed correctly in order to have complemented the RNAiPIII transgenic line. The NtCp and PIIICA constructs did not complement the reduced PELPIII mRNA levels, despite levels of mRNA similar to those of the constructs that did complement the reduced normal PELPIII levels. The PELPIII NTD with the PELPIII or TTS CTD was essential to complement RNAiPIII.

## Discussion

### PELPIII acts in species-specific PTG inhibition

The PELPIII AGP is essential for species-specific interspecific PTG inhibition [10]. Reduction of endogenous PELPIII (S plus T) mRNA levels in the RNAiPIII transgenic line resulted in increased *N. obtusifolia* PTG relative to normal styles but did not alter PTG of thirteen other *Nicotiana* species, including *N. repanda* (Additional file 1). It was previously shown that pollination of styles of antisense plants with reduced PELPIII mRNA levels in *N. tabacum* ‘Petite Havana’ SR1 resulted in longer PTG of both *N. obtusifolia* and *N. repanda* relative to normal ‘Petite Havana’ SR1styles [10, 11]. Our results were similar to those of Eberle [10] with reduced PELPIII in the RNAiPIII transgenic line and increased *N. obtusifolia* PTG (Fig. 4). However, inhibition of *N. repanda* PTG was not reduced in the RNAiPIII transgenic line, in contrast to the results of Eberle [10] using the antisense PELPIII in ‘Petite Havana’ SR1. The difference is likely due to the different genotypes that were used in the two studies, ‘Samsun’ vs. ‘Petite Havana’ SR1. Transgenic lines may have different levels of PELPIII or may have differences in other genes that regulate PTG. The TT-ablated transgenic line of ‘Samsun’ that lacks a mature TT and most of its associated proteins, also reduced the inhibition of *N. obtusifolia* and *N. repanda* PTG [25]. *N. obtusifolia* and *N. repanda* grew 21.5 and 18.8 mm in ‘Samsun’ lacking a mature TT, respectively, and 12.2 and 4.3 mm in normal plants, respectively. The increased PTG in the TT-ablated line strengthens the hypothesis that other TT proteins are involved in *N. repanda* PTG inhibition. The PTG of thirteen *Nicotiana* species did not differ between normal and the RNAiPIII line, suggesting that PTG of these species are not affected by the presence or reduction of PELPIII. PELPIII acts in a species-specific manner in PTG inhibition and that different mechanisms and factors are involved in regulation of *N. obtusifolia* PTG.

### The PELPIII NTD is required for *N. obtusifolia* pollen tube growth inhibition

PELPIII is essential for the inhibition of *N. obtusifolia* PTG (Fig. 4) [10]. Among AGPs and *Nicotiana* species, the PELPIII NTD has the highest level of polymorphism compared to the PELPIII CTD [15]. This led to the hypothesis that the NTD is the domain responsible for *N. obtusifolia* PTG inhibition. Crossing RNAiPIII with plants expressing the PIIICtrl gene construct shows this strategy can test for complementation. The PIIICtrl_RNAiPIII_-1 and -2 transgenic lines complemented the reduced level of PELPIII as measured by the inhibition of *N. obtusifolia* PTG in the RNAiPIII background (Fig. 4). These results validate the strategy to use RNAi specific to the PELPIII 3’UTR region to eliminate its expression, followed by testing the activity of variant PELPIII gene constructs with a novel 3’UTR. The inhibition of *N. obtusifolia* PTG by the PIIICtrl gene shows that the construct’s timing and level of PELPIII accumulation with the *sp41* promoter and 7S 3’UTR was sufficient to complement the reduction of PELPIII mRNA by RNAiPIII. The control gene construct only expresses the PELPIII-S coding sequence (lacks PELPIII-T coding sequence), showing that a single PELPIII-S gene can complement the reduction of both S and T PELPIII.

The NpCt_RNAiPIII_ line was used to test whether the TTS CTD functions similarly to the PELPIII CTD. The NpCt_RNAiPIII_-1 and -2 transgenic lines had variant NpCt mRNA levels that were significantly different and 3.5-fold lower than total endogenous PELPIII mRNA in normal plants (Fig. 3). The variant NpCt construct complemented the RNAiPIII transgenic line as measured by the inhibition of *N. obtusifolia* PTG. These results suggest that in the NpCt_RNAiPIII_ transgenic lines, the NpCt variant protein accumulates in an active form at a sufficient level to inhibit *N. obtusifolia* PTG. The lack of a PELPIII specific activity measurement for normal or variant PELPIII in the *N. tabacum* styles does not refute the conclusion that the NpCt gene construct complements the reduction of PELPIII. Therefore, the PELPIII and TTS CTD sequences provide a similar structure and function to PELPIII, when combined with the PELPIII NTD.

The NtCp gene construct was used to test if the TTS NTD can substitute for the PELPIII NTD with the PELPIII CTD. The transgenic lines NtCp_RNAiPIII_-1 and -2 had levels of NtCp mRNA were equal to or greater than the total endogenous PELPIII levels in normal *N. tabacum* and not significantly different or higher than the levels of NpCt mRNA in the NpCt_RNAiPIII_ lines (Fig. 3). However, the NtCp gene construct failed to complement the RNAiPIII background, showing no inhibition of *N. obtusifolia* PTG (Fig. 4). The NTD has a high level of diversity among AGPs and is predicted to be highly glycosylated [15]. While the TTS NTD is predicted to have four glycosylation sites, the PELPIII NTD is predicted to have eight glycosylation sites [15]. The TTS facilitates *N. tabacum* self-PTG and is deglycosylated during PTG and reducing TTS slowed self PTG [3, 4]. In contrast, reduction of PELPIII mRNA levels had no effect on self PTG and PELPIII is not deglycosylated during PTG [12]. Differences in the glycosylation pattern of the PELPIII and TTS AGPs or their deglycosylation during PTG may be associated with the inhibition of *N. obtusifolia* PTG. The failure of the NtCp variant to inhibit *N. obtusifolia* PTG confirms the essential nature of the PELPIII NTD for normal PELPIII activity.

The mechanism through which the TTS and PELPIII NTDs act differently in the regulation of PTG is not known. However, the AGPs may act through interactions with other proteins to regulate PTG. For self-incompatibility, 120 K, SLF (*S*-locus F-box gene), NaTrxh (thioredoxin H) and SBP1 (*S*-RNase binding protein 1) interact with *S*-RNase and are required for self-incompatibility in *N. alata* [26, 27]. PELPIII may interact with yet unidentified proteins that are essential for the regulation of PTG. The NpCt gene construct’s complementation of reduced PELPIII suggests that proteins interacting with the endogenous PELPIII also interact with the NpCt PELPIII and that the CTD of PELPIII or TTS has similar structure and function. A significant difference between the TTS and PELPIII NTDs is their amino acid lengths. The PELPIII NTD is 2.3 times (164 amino acid) longer than the TTS NTD (Fig. 1), of which the vast majority are proline residues. Proline has an important role in AGP structure because it is post-translationally modified to hydroxyproline where glycosylation can occur [13, 28]. Since most of the polymorphisms between PELPIII and TTS residues are in the NTD and this region may form interactions with other proteins, it is reasonable to conclude that the PELPIII NTD is a major contributor to the differential activity of PELPIII and TTS.

Cysteine forms disulfide bonds and plays a role in protein folding, stability and interaction with other proteins [21, 22]. PELPIII has a unique cysteine in its NTD compared to TTS and 120 K AGPs. Because the unique NTD cysteine may be critical for PELPIII function, it was mutated to alanine, one of the simplest amino acids and with a non-reactive side-chain used in many studies as an amino acid replacement [29–31]. The PIIICA construct in the transgenic lines PIIICA_RNAiPIII_-1 and -2 did not complement RNAiPIII and *N. obtusifolia* PTG was not significantly different from that in the RNAiPIII line (Fig. 4) despite having PIIICA mRNA accumulation levels that were similar to endogenous PELPIII levels in normal plants. The cysteine in the PELPIII NTD may form disulfide bonds and stabilize PELPIII as in potato ADP-glucose phosphorylase, where a single mutation of cysteine to alanine or serine resulted in reduced heat-stability and reduced activity [32]. The mutation of cysteine to alanine in the PELPIII NTD results in defects resulting in the lack of *N. obtusifolia* PTG inhibition.

## Conclusions

PELPIII accumulation in the mature TT of the *N. tabacum* style acts in a species-specific manner to inhibit PTG of *N. obtusifolia.* The PELPIII NTD can be combined with either the PELPIII CTD or TTS CTD for normal PELPIII activity as measured by *N. obtusifolia* PTG inhibition. The failure of NtCp and PIIICA gene constructs to complement the reduction of PELPIII mRNA suggests that the PELPIII NTD has a specific structure that is essential for its function in interspecific incompatibility. Future studies on the mechanism of PELPIII NTD inhibition of PTG should focus on the polymorphisms between the PELPIII and TTS NTDs and how they may result in distinct and species-specific PTG regulation.

## Methods

### Plant material

Seeds of *Nicotiana* species were sowed and grown in Metro-Mix 360 medium (Sun Gro Horticulture, Massachusetts, USA) in a greenhouse at 21 °C under a photoperiod of 14 h day/10 h night, with supplemental light from 400 W HPS high pressure sodium lamps [15, 25]. Transgenic lines were produced in *N. tabacum* ‘Samsun’. Normal plants used as a control were male-sterile transgenic plants without changes to pistil morphology or PTG regulation, obviating the need for emasculation before pollinating [2, 33].

### PELPIII and TTS amino acid sequences comparison

*Nicotiana tabacum* is an allotetraploid from the hybridization of *N. sylvestris* (S) and *N. tomentosiformis* (T) and has two PELPIII and TTS genes that share similarities with the ancestral *N. sylvestris* (PELPIII-S and TTS-S; Fig. 1) and *N. tomentosiformis* (PELPIII-T and TTS-T; Fig. 1) genes [15]. The *N. tabacum* gene sequences for PELPIII-S and -T (accession Z14019.1 and Z14015.1, respectively) and TTS-S and -T (accession Z16403.1 and Z16404.1, respectively) were obtained from NCBI (http://www.ncbi.nlm.nih.gov/). The Z16403.1 sequence from TTS-S has an additional cytosine from a sequencing error at a position 687 bp from the start codon causing a frame shift. Deletion of the cytosine resulted in an open reading frame [15] and was used in this study. The NTD and CTD domains, as defined by Hancock [14], were compared using Clustal Omega [34].

### Gene constructs

To test the hypothesis that the NTD is essential for determining PELPIII function in the inhibition of interspecific PTG the PELPIII mRNA level was reduced using 3’UTR RNA interference (RNAiPIII; Additional file 2). All transgenes (RNAiPIII and PELPIII variants) were cloned into the sterility gene construct used by Gardner [33] by substitution of sterility gene. A standard Gibson reaction using gene blocks synthesized by Integrated DNA technologies (IDT, Iowa, USA) or PCR amplicons was used to assemble constructs (New England Biolabs, Massachusetts, USA) [35]. Use of the 3’UTR sequences for RNAi allowed complementation with variant PELPIII constructs produced with the 7S 3’UTR from the seed storage protein gene of soybean (7S) [36] and a modified *sp41* TT-specific promoter (Additional file 3) [33, 37]. The use of the 7S 3’UTR in the variant PELPIII gene constructs avoids reduction of a variant PELPIII mRNA by the RNAiPIII construct. The *sp41* promoter used by Gardner [33] had two ATGs followed by a TATA box at positions − 15 and 31 that has a potential to reduce translation efficiency [37]. To prevent translational attenuation, the thymine was mutated to adenine in both ATGs. The TT-specific modified *sp41* promoter was synthesized by IDT (Additional file 3) and introduced into the sterility gene construct after *Bam*HI restriction enzyme digestion followed by Gibson reaction to assemble all gene blocks (New England Biolabs, Massachusetts, USA) [35]. Digestion with *Nco*I removed the *sp41*: barnase gene from the sterility gene construct [33], which was replaced with the RNAiPIII or a variant PELPIII genes containing the modified *sp41* promoter and the 7S seed storage 3’UTR [36].

#### RNAiPIII gene construct

The RNAiPIII gene construct contains sense and antisense 3’UTRs sequences from *N. tabacum* PELPIII-S and PELPIII-T genes to reduce mRNA of both genes (Additional file 2). The PELPIII-S 3’UTR sequence used was from 1376 to 1469 (Z14019.1) and PELPIII-T 3’UTR is from 1254 to 1347 (Z14015.1). The PELPIII-S and PELPIII-T 3’UTR sequences used were 94 nucleotides in length and differed by four nucleotides (Additional file 4). The RNAiPIII gene construct was generated using three gene blocks [38]. Among a number of independent transgenic lines carrying the RNAiPIII construct, a single line was selected with low PELPIII-S and -T mRNA accumulation and carried a single transgene insert.

The variant PELPIII gene constructs contained the PELPIII-S or TTS-S sequences. The TTS-S gene has greater mRNA accumulation than the TTS-T gene in *N. tabacum* (referred to as TTS-1 and TTS-2, respectively in Quiapim [39] and has 92.9% amino acid identity to TTS-T. The PELPIII-S has greater mRNA accumulation than PELPIII-T and has 89.3% amino acid identity to PELPIII- T (Figs. 1 and 3).

#### PIIICtrl gene construct

The PELPIII control gene construct (PIIICtrl) was used to test if expressing the PELPIII gene using the sp41 promoter, the 7S 3’UTR and a normal coding sequence could complement the loss of PELPIII as measured by the relative inhibition of *N. obtusifolia* PTG. To produce the control PELPIII gene construct, three gene blocks were used (Additional file 5). The PELPIII coding sequence was taken from bases 11 to 1291 from accession number Z14019.1.

#### PIIICA gene construct

The PELPIII NTD has an additional cysteine compared to the TTS NTD (Fig. 1). To determine if the cysteine at position 156 in the PELPIII NTD is essential for PELPIII function, it was mutated to alanine and the variant gene construct (PIIICA) was used to complement the RNAiPIII line. The PIIICA gene construct was generated by amplifying PELPIII from the PIIICtrl construct using two primer pairs (Additional files 6 and 7) to generate two amplicons. The primers PIIIc-aM2-R and PIIIc-aM3-F were used to introduce the mutation into the PELPIII sequence by PCR.

#### NpCt gene construct

The NpCt gene construct contains the PELPIII NTD (Np) and the TTS CTD (Ct). The PELPIII and TTS CTDs share six cysteines in common and are more conserved relative to the PELPIII and TTS NTDs (Fig. 1). The NpCt gene construct was generated to test whether the TTS CTD can substitute for the PELPIII CTD and complement the loss of PELPIII function in the RNAiPIII transgenic line as measured by inhibiting *N. obtusifolia* PTG*.* The NpCt gene construct was generated by amplicon and gene block synthesis. One pair of primers (Additional file 6) amplified the PELPIII NTD from the PIIICtrl construct and the TTS CTD was synthesized as one gene block (Additional file 8). The PELPIII NTD corresponds to nucleotide positions 11 to 877 from accession Z14019.1 and the TTS CTD was synthesized from 382 to 793 from accession Z16403.1.

#### NtCp gene construct

The NtCp gene construct contains the TTS NTD (Nt) and the PELPIII CTD (Cp) and was used to assess whether this combination could complement the RNAiPIII transgenic line. The NtCp gene construct was generated by PCR amplification and gene block synthesis. One pair of primers (Additional file 6) amplified the TTS NTD from TTS corresponding to position 19 to 381 from NCBI Z16403.1 (Additional file 9) and the PELPIII CTD was synthesized from 878 to 1291 from accession Z14019.1 (Additional file 10).

All genes were sequenced to confirm construct assembly. The gene constructs containing the *sp41* modified promoter and 7S 3’UTR were digested with *Not*I and ligated with the plant transformation vector pMON886 [36]. The resulting vectors were introduced into *Agrobacterium tumefaciens* strain LBA4404 by electroporation. Leaf disks of *N. tabacum* ‘Samsun’ were transformed as described by Gardner [33] with the exception that selection medium contained 300 mg/l of cefotaxime and 100 mg/l of kanamycin. Confirmation of transformation in regenerated plants was done by construct-specific PCR (Additional file 6). Copy number of the transgenes were evaluated by selfing plants, determining the segregation ratios of kanamycin resistance and only transgenic lines segregating 3:1 for kanamycin resistance: kanamycin sensitive were used for further analysis. Two independent transgenic lines, each with a single transgene insert (heterozygous) were selected for crossing to the T0 heterozygous RNAiPIII line to produce the F1 heterozygous control and complementation lines used in this analysis.

### Quantitative real-time PCR analysis

Quantitative Reverse Transcriptase PCR (qRT-PCR) was performed using a SYBR green reagent system in a CFX96 Touch™ Real-Time PCR Detection System (Bio-Rad, California, USA) to determine steady-state mRNA levels in the style. Styles were collected from four flowers from the same plant at stage 12 (open and mature flowers) and stored at − 80 °C [40]. The four styles were ground together and represent one biological sample. Total RNA was extracted using a ZR Plant RNA MiniPrep (Zymo Research, Californa, USA) and the RNA concentration was measured using a Nanodrop Spectrophotometer (Thermo Scientific, Massachusetts, USA). 3 μg of RNA was treated with 1 μl of DNase I (2 U/μl; RNase-free, New England Biolabs, Massachusetts, USA) and 0.5 μl of RNaseOUT (Thermo Fisher Scientific, Massachusetts, USA) for 30 min at 37 °C and inactivated for 10 min at 70 °C.The DNase I-treated RNA was tested for genomic DNA contamination by PCR. The PCR reactions were prepared using 12.5 μl GoTaq Green Master Mix 2X (Promega, Wisconsin, USA), 25 μM of primer PIII-F, 25 μM of primer PIII-R (Additional file 6) and 261 ng of RNA with 40 cycles. The PCR products were separated by electrophoresis in 0.7% agarose gels and visually scored for the presence of amplified genomic DNA. cDNA synthesis was performed using 3 μg of DNase-treated RNA, 1 μl of M-MLV reverse transcriptase (200 U/μl, Promega, Wisconsin, USA), 4 μl of 5X reaction buffer (Promega, Wisconsin, USA), 10 mM dNTP Mix (GenScript, New Jersey, USA), 1 μl of 500 μg/mL Oligo (dt)_12–18_, 2 μl of 0.1 M DTT in a total reaction volume of 20 μl. The qRT-PCR reaction was performed using iTaq™ Universal SYBR® Green Supermix (10 μl; BioRad, California, USA), gene-specific primers (400 nM each, Additional file 6), cDNA template (90 ng) and water for a 20 μl total reaction volume.

The endogenous (S plus T) and variant PELPIII mRNA levels were determined for complemented plants resulting from the cross of RNAiPIII and a control or variant-expressing transgenic line. qRT-PCR analyses were performed on transgenic lines that were confirmed by PCR to contain both the RNAiPIII and the control or a variant gene construct to measure endogenous PELPIII-S, PELPIII-T and variant PELPIII mRNA levels. The relative quantification was calculated by the dCq method (delta threshold cycle, BioRad) [41] using actin (accession GQ281246.1) as a reference gene. The dCq was calculated between actin and the target gene for each of two technical replicates and then averaged across three biological replicates. The mRNA levels of PELPIII-S and T were measured separately. To sum the levels of PELPIII-S and T, the qRT-PCR data was first normalized to actin levels and a dCq value was calculated for S and T. The S and T dCq values were then converted to linear amounts by 2^dCq. The linear S and T values were summed and then converted back to log base 2 values (dCq) for Fig. 3. The fold change was calculated by dividing the mRNA levels being compared after converting from log_2_ to linear values. Analysis of variance (ANOVA) was performed and Tukey’s HSD test at α = 0.05 compared the dCq means among transgenic lines. Plants confirmed to have a low level endogenous PELPIII and a normal level of control or variant PELPIII mRNA were used for the PTG analysis.

### Pollen tube growth measurements and data analysis

Mature stage 12 flowers were pollinated with 20,000 pollen grains/μl by the method described by Gardner [33] and Eberle [2] with intact stigmas.. Paired t-tests at α = 0.05 were used to compare PTG in normal and RNAiPIII styles, ANOVAs followed by Tukey’s Honest Significant Difference (HSD) at α = 0.05 to compare accumulation of mRNA levels in different genotypes, or Dunnett’s test at α = 0.05 to compare PTG in complementation lines styles compared to the control normal style. A Chi square test was used to test for 3:1 segregation (Kanamycin resistant: Kanamycin susceptible).

## Additional files

Additional file 1: Evaluations of *Nicotiana* species PTG in normal *N. tabacum* ‘Samsun’ and the RNAiPIII transgenic line. Five pollinations, replicated twice in time, were performed for each pollen-style combination. Pollen tubes were measured from the stigma to the end of the PTG front 40 h post pollination [10]. Different letters indicate a significant difference in PTG between normal and RNAiPIII plants as determined by a paired t-tests at α = 0.05. (DOCX 69 kb)

Additional file 2: Gene blocks for RNAiPIII gene construct. Three gene blocks were used to generate the RNAiPIII construct. Gene block 1 contains: NcoI restriction site (black rectangle), sense 3’UTR of the PELPIII-S and -T (light blue and dark blue, respectively), 5′ end of the Pdk intron (gray); Gene block 2 contains the Pdk intron sequence continued from block 1 (gray); Gene block 3 contains: 3′ end of the Pdk intron (gray); antisense 3’UTR of the PELPIII-T and -S (dark blue and light blue, respectively), *Nco*I restriction site (black rectangle). All gene blocks have overlapping regions (bold) designed for Gibson assembly. PELPIII-S and -T sequences were taken from accession Z14019.1 and Z14015.1, respectively. (DOCX 26 kb)

Additional file 3: The TT-specific SP41 modified promoter gene block sequence used in all gene constructs (Fig. 1). The promoter gene block contains the mutations of two thymines to adenine in the original *sp41*sequence (from position 1027 to 1455; accession X81560.1) followed by a TATA box (violet shading). Thymines mutated to adenosine are at positions 1306 and 1323 (blue shading). Bolded sequences represent overlapping regions and restriction enzyme sites for cloning. Black rectangles show the *Bam*HI restriction sites used to insert the gene block into the sterility gene construct plasmid [33] (DOCX 13 kb)

Additional file 4: Comparisons of the 3’UTR of PELPIII-S and -T used in the RNAiPIII construct. The 3’UTR sequences of PELPIII-S and -T (accession Z14019.1 and Z11015.1, respectively) have 95.7% nucleotide identity. Asterisks show identical nucleotides. Gray-shaded letters represent the four nucleotide differences between the genes. Numbers between parentheses show the sequence positions. (DOCX 13 kb)

Additional file 5: Gene blocks used to generate the PIIICtrl gene. Gene block 1 contains the start codon ATG (green shading), *NcoI* restriction enzyme site (black rectangle) and part of the PELPIII-S NTD; Gene block 2 contains parts of the PELPIII-S NTD and CTD; Gene block 3 contains the remainder of the PELPIII-S CTD, stop codon TGA (gray shading) and *NcoI* restriction enzyme site (black rectangle). All gene blocks have an overlapping region designed for Gibson assembly (bold). The PELPIII-S sequence used for the PIIICtrl gene construct is NCBI accession Z14019.1. (DOCX 15 kb)

Additional file 6: Primers used to detect gene constructs, perform PCR and qRT-PCR. PCR of *N. sylvestris* and *N. tomentosiformis* genomic DNA was performed to test the construct- and gene-specificity of each primer pair. In those PCR reactions, amplification was only detected by the appropriate primers and no PELPIII was amplified by the S-specific primer pair with *N. tomentosiformis* genomic DNA and no PELPIII was amplified by the T-specific primer pair with *N. sylvestris* genomic DNA. (DOCX 21 kb)

Additional file 7: Amplicons used to generate the PIIICA gene construct. Amplicon 1 contains: *Nco*I restriction enzyme site (black rectangle), start codon (green shading), part of the PELPIII NTD including the cysteine mutated to alanine (blue shading); Amplicon 2 contains: part of the PELPIII NTD including the cysteine mutated to alanine (blue shading), stop codon (gray) and NcoI restriction enzyme site (black rectangle). The amplicons have overlapping regions designed for Gibson assembly (bold) and were amplified from PIIICtrl (accession Z14019.1). Amplicons 1 and 2 have the primer sequences used to mutate cysteine to alanine (underlined and italic). (DOCX 15 kb)

Additional file 8: Amplicon and gene block used to generate the NpCt gene. Amplicon 1 NpCt contains: *Nco*I restriction enzyme site (black rectangle), a start codon (green shading) and the PELPIII NTD amplified from PIIICtrl (accession Z14019.1); Gene block 1 NpCt contains: the TTS CTD, the stop codon TAA (gray shading) and a NcoI restriction enzyme site (black rectangle). The amplicons have overlapping regions designed for Gibson assembly (bold). Primers used to amplify PELPIII NTD are underlined and italicized. (DOCX 15 kb)

Additional file 9: Gene blocks used to generate the TTS NTD and the TTS CTD. Gene block 1 contains start codon ATG (green shading), *Nco*I restriction enzyme site (black rectangle) and part of the TTS-S NTD; Gene block 2 contains part of the TTS-S NTD, the TTS-S CTD, the stop codon TAA (gray shading) and the *Nco*I restriction enzyme site (black rectangle). The gene blocks have overlapping regions designed for Gibson assembly (bold). The TTS-S sequence used for gene constructs is NCBI accession Z16403.1. (DOCX 14 kb)

Additional file 10: Amplicon and gene block used to generate the NtCp gene. Amplicon 1 NtCp contains: *Nco*I restriction enzyme site (black rectangle), start codon (green shading) and TTS NTD amplified from TTS-S (accession Z16403.1); Gene block 1 NtCp contains: PELPIII CTD, stop codon TGA (gray shading) and *Nco*I restriction enzyme site (black rectangle). The amplicons have overlapping regions designed for Gibson assembly (bold). Primer sequences used to amplify TTS NTD are underlined and italicized. (DOCX 15 kb)

## Abbreviations

120 K: 120KDa glycoprotein
AGP: Arabinogalactan protein
Cp: PELPIII CTD
Ct: TTS CTD
CTD: C-terminal domain
IDR: Intrinsically disordered regions
Np: PELPIII NTD
NpCt: Gene construct with PELPIII NTD and TTS CTD
Nt: TTS NTD
NtCp: Gene construct with TTS NTD and PELPIII CTD
NTD: N-terminal domain
PELPIII: Class III pistil extensin-like protein
PIIICA: PELPIII with cysteine in NTD position 156 mutated to alanine
PIIICtrl: PELPIII control gene construct
PTG: Pollen tube growth
RNAiPIII: PELPIII RNA interference construct
-S: *N. sylvestris* as genome donor;
-T: *N. tomentosiformis* as genome donor;
TT: Transmitting tissue
TTS: Transmitting tissue-specific
