# Supplementary figures and images for: Structure and function of class III pistil-specific extensin-like protein in interspecific reproductive barriers
Source: BMC Plant Biol. 2019 Mar 29;19:118. doi: 10.1186/s12870-019-1728-8 (PMC6440088; doi:10.1186/s12870-019-1728-8)

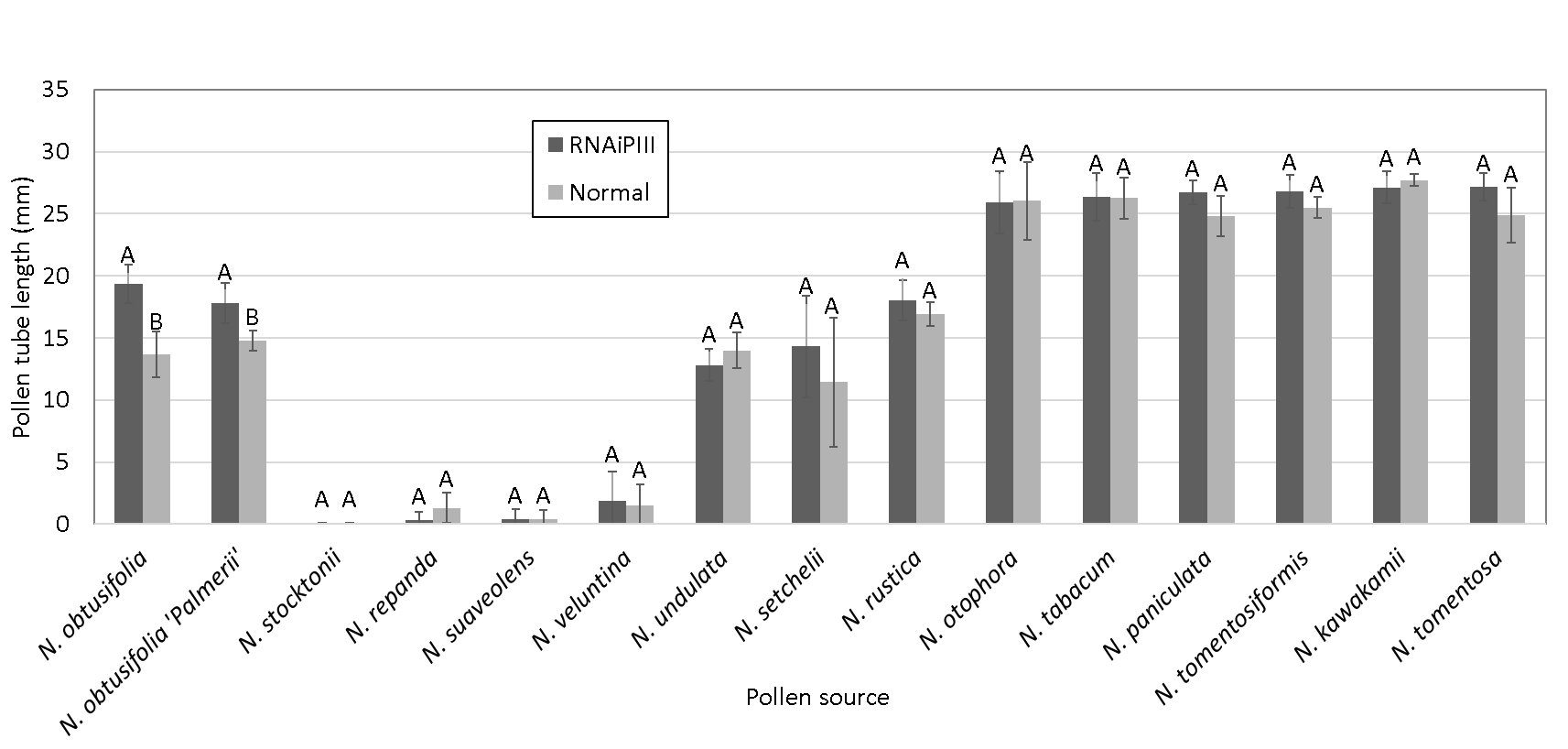

Supplement: Supplementary file 1 — Evaluations of Nicotiana species PTG in normal N. tabacum ‘Samsun’ and the RNAiPIII transgenic line. Five pollinations, replicated twice in time, were performed for each pollen-style combination. Pollen tubes were measured from the stigma to the end of the PTG front 40 h post pollination [10]. Different letters indicate a significant difference in PTG between normal and RNAiPIII plants as determined by a paired t-tests at α = 0.05. (DOCX 69 kb) [file 12870_2019_1728_MOESM1_ESM.docx]
