# Supplementary material for: Structure and function of class III pistil-specific extensin-like protein in interspecific reproductive barriers
Source: BMC Plant Biol. 2019 Mar 29;19:118. doi: 10.1186/s12870-019-1728-8 (PMC6440088; doi:10.1186/s12870-019-1728-8)
Supplement: Supplementary file 2 — Gene blocks for RNAiPIII gene construct. Three gene blocks were used to generate the RNAiPIII construct. Gene block 1 contains: NcoI restriction site (black rectangle), sense 3’UTR of the PELPIII-S and -T (light blue and dark blue, respectively), 5′ end of the Pdk intron (gray); Gene block 2 contains the Pdk intron sequence continued from block 1 (gray); Gene block 3 contains: 3′ end of the Pdk intron (gray); antisense 3’UTR of the PELPIII-T and -S (dark blue and light blue, respectively), NcoI restriction site (black rectangle). All gene blocks have overlapping regions (bold) designed for Gibson assembly. PELPIII-S and -T sequences were taken from accession Z14019.1 and Z14015.1, respectively. (DOCX 26 kb) [file 12870_2019_1728_MOESM2_ESM.docx]

**Gene block 1 (447 bp)**

**tgcttAGTATCAAGCTTATCGATAGATCTCCATGG**GTTCTATAGTTTATACAAGGAGACAGAAAACTTTGTACCACTATACAGAAATCAAATcAGTCGgAAAAtTCAAAATCGAAcTTATGAAAACTGAGTTCTATAGTTTATACAAGGAGACAGAAAACTTTGTACCACTATACAGAAATCAAATGAGTCGCAAAAGTCAAAATCGAATTTATGAAAACTGActcgaggaattcggtaccccaattggtaaggaaataattattttcttttttccttttagtataaaatagttaagtgatgttaattagtatgattataataatatagttgttataattgtgaaaaaataatttataaatatattgtttacataaacaacatagtaatgtaaaaaaatatgacaagtgatgtgtaa**gacgaagaagataaaagttgagagtaagta**

**Gene block 2 (541 bp)**

**Gacgaagaagataaaagttgagagtaagta**Tattatttttaatgaatttgatcgaacatgtaagatgatatactagcattaatatttgttttaatcataatagtaattctagctggtttgatgaattaaatatcaatgataaaatactatagtaaaaataagaataaataaattaaaataatatttttttatgattaatagtttattatataattaaatatctataccattactaaatattttagtttaaaagttaataaatattttgttagaaattccaatctgcttgtaatttatcaataaacaaaatattaaataacaagctaaagtaacaaataatatcaaactaatagaaacagtaatctaatgtaacaaaacataatctaatgctaatataacaaagcgcaagatctatcattttatatagtattattttcaatcaacattcttattaatttctaaataatacttgtagttttattaacttctaaatggattgactattaattaa**atgaattagtcgaacatgaataaacaaggt**

**Gene block 3 (349bp)**

**atgaattagtcgaacatgaataaacaaggt**aacatgatagatcatgtcattgtgttatcattgatcttacatttggattgattacagttgggaaattgggttcgaaatcgataagcttggatcctctagaTCAGTTTTCATAAATTCGATTTTGACTTTTGCGACTCATTTGATTTCTGTATAGTGGTACAAAGTTTTCTGTCTCCTTGTATAAACTATAGAACTCAGTTTTCATAAgTTCGATTTTGAaTTTTcCGACTgATTTGATTTCTGTATAGTGGTACAAAGTTTTCTGTCTCCTTGTATAAACTATAGAAC**CCATGGcatgGGTACCccgGGATCCCGTCCT**
