# Supplementary material for: Structure and function of class III pistil-specific extensin-like protein in interspecific reproductive barriers
Source: BMC Plant Biol. 2019 Mar 29;19:118. doi: 10.1186/s12870-019-1728-8 (PMC6440088; doi:10.1186/s12870-019-1728-8)
Supplement: Supplementary file 3 [file 12870_2019_1728_MOESM10_ESM.docx]

Amplicon 1 NtCp – contains TTS NTD (424 bp)

*TGCTTAGTATCAAGCTTATCGATAGATCTCCATGGCAAAGGCCCTTGTTC*TTTTTCAGCTTTCAGTTTTAT
TACTTAGCTCATTCACAGTAGTTCTTAGCCAGGAGGAAGGCATTGGGGGTTGGTTTACCACCAAACATCAT
GACCACCTTTCACCAGCTCAAGCTCCTAAGCCTCACAAAGGCAACCACCACCCCAAACATTCCCCAGCCCC
TTCACCAACTAAGCCTCCCACTTATAGCCCATCGAAACCACCAGTTAAACCACCGGTTAAACCACCAACTA
AGGCTCCCACTTATAGCCCATCAAAACCACCAGCTAAGCCACCAGTTAAACCACCAACACCAACACCATCA
CCTTATCCTGCTCCTG*CTCCTATTACTAGGAAACCTGTA****ATCGTCAGTGGCCTTGTTTATTGTAAATCC***

Gene block 1 NtCp – contains PELPIII CTD (445 bp)

ATCGTCAGTGGCCTTGTTTATTGTAAATCCTGCAACAGCTATGGGGTTCCCACTCTGCTCAACGCCTCCCT
ACTCCAAGGAGCTGTTGTGAAACTAATTTGCTACGGAAAAAAAACAATGGTTCAATGGGCCACGACAGACA
ACAAAGGTGAGTTTCGGATCATGCCCAAATCTTTAACCACAGCAGATGTTGGCAAGTGCAAGGTATATTTA
GTGAAATCACCAAATCCAAATTGCAATGTCCCAACAAATTTCAATGGTGGAAAATCTGGTGGTTTATTGAA
GCCTCTCCTACCACCTAAACAACCGATTACCCCTGCCGTTGTCCCTGTCCAACCACCCATGTCTGATTTAT
ATGGTGTTGGACCTTTTATATTTGAAGCCTCCAGCAAAATGCCATGCGATAAGAATTGACCATGGCATGGG
TACCCCGGGATCCCGTCCT
