# Supplementary material for: Structure and function of class III pistil-specific extensin-like protein in interspecific reproductive barriers
Source: BMC Plant Biol. 2019 Mar 29;19:118. doi: 10.1186/s12870-019-1728-8 (PMC6440088; doi:10.1186/s12870-019-1728-8)
Supplement: Supplementary file 4 [file 12870_2019_1728_MOESM3_ESM.docx]

AAACAATCATCATGCAGATATATTAAATGGGATCCATTTTTCCTGTACAATTCCATACCTCCATCACTCTTTAGTGGAA
CTTGTGCAAAGTTTTCTTTATTTATGTCAAAATTGCACTATTATGCCATGAAATGCGTCACTTGGTATGCTTGGATTCT
TTACATTTCTGTAGTAGTATTTGTTTTTTAAGTTAAGTCATTGCCTAAGACAGCTCCATCTTCTATTGCTTGCCTATAT
AAGAAGCAATAGAATGCTTCCTTAATGTACAATTCTAACCCAaGGTTAACTTCTTAACAaGTGTTACTAAGCCAAACTG
AATCCCTTTCCGAGGAGGGGATTCCATTATCTTTCCAACTGAACTTAAGATTGACCTAACTGCCTCGTTCTTGAAACTG
CACTCTCTTTCTTAATTGACCTTCTTGC**TTAGTATCAAGCTTATCGATAGATCTCCATGGCATGGGTACCCCGGGATCC**
CGTCCTTTGTCTTCAATTTTGAGGG
