# Supplementary material for: Structure and function of class III pistil-specific extensin-like protein in interspecific reproductive barriers
Source: BMC Plant Biol. 2019 Mar 29;19:118. doi: 10.1186/s12870-019-1728-8 (PMC6440088; doi:10.1186/s12870-019-1728-8)
Supplement: Supplementary file 5 [file 12870_2019_1728_MOESM4_ESM.docx]

```
3'UTRPELPIII-S (1376) GTTCTATAGTTTATACAAGGAGACAGAAAACTTTGTACCACTATACAGAAATCAAATcAG
3'UTRPELPIII-T (1254) GTTCTATAGTTTATACAAGGAGACAGAAAACTTTGTACCACTATACAGAAATCAAATGAG
                      ********************************************************* **

3'UTRPELPIII-S        TCGgAAAAtTCAAAATCGAAcTTATGAAAACTGA (1469)
3'UTRPELPIII-T        TCGCAAAAGTCAAAATCGAATTTATGAAAACTGA (1347)
                      *** **** *********** *************
```
