# Supplementary material for: Structure and function of class III pistil-specific extensin-like protein in interspecific reproductive barriers
Source: BMC Plant Biol. 2019 Mar 29;19:118. doi: 10.1186/s12870-019-1728-8 (PMC6440088; doi:10.1186/s12870-019-1728-8)
Supplement: Supplementary file 6 [file 12870_2019_1728_MOESM5_ESM.docx]

Gene block 1 PIIICtrl (452 bp)
TGCTTAGTATCAAGCTTATCGATAGATCTCCATGGCTGTTATTATTTCATCGAAGGTCCTACTTATACAAC
TTTTCGTTCTAGTACTTGGCTCATTCTCAAAGCTCAGCCATGGGGAGCTGTGGCTGGAACTCCCATTGCCT
TTCGACTGGCCACCAGCGGAGATCCCATTGCCGGATATCCCATCGCCTTTCGATGGGCCTACATTCGTGCT
ACCGCCACCGTCACCATTGCCATCACCACCTCCGCCATCTCCATCACCACCTCCGCCATCACCATCACCAC
CTCCTCCGTCGACAATACCACTTATTCCTCCTTTTACCGGCGGCTTTTTGCCTCCTTTGCCCGGCTCTAAG
CTTCCTGATTTTGCCGGCCTTTTGCCTCTGATTCCCAACTTACCTGATGTGCCTCCCATAGGTGGTG**GTCC**
ACCTGTTAACCAGCCAAAGCCGTCGT

Gene block 2 PIIICtrl (500 bp)
GTCCACCTGTTAACCAGCCAAAGCCGTCGTCACCATCACCCCTAGTTAAGCCGCCACCACCACCACCTTCA
CCATGTAAGCCATCACCACCTGATCAGTCTGCAAAGCAACCACCACAACCTCCTCCAGCAAAACAACCATC
ACCTCCACCTCCTCCACCACCAGTTAAGGCACCATCTCCATCTCCAGCAAAGCAACCACCACCTCCTCCAC
CGCCAGTTAAGGCACCATCTCCATCTCCAGCCACTCAGCCACCTACAAAGCAACCGCCACCGCCGCCACGT
GCTAAGAAATCGCCTCTTCTACCTCCTCCGCCACCAGTTGCTTATCCGCCAGTTATGACACCATCTCCATC
ACCGGCTGCTGAGCCACCTATTATAGCACCATTTCCATCACCACCAGCGAATCCACCCCTCATTCCCCGTC
GACCAGCACCACCAGTAGTTAAGCCGCTTCCACCTTTGGGGAAG**CCCCCAATCGTCAGTGGCCTTGTTTAT**
TGT

Gene block 3 PIIICtrl (451 bp)
CCCCCAATCGTCAGTGGCCTTGTTTATTGTAAATCCTGCAACAGCTATGGGGTTCCCACTCTGCTCAACGC
CTCCCTACTCCAAGGAGCTGTTGTGAAACTAATTTGCTACGGAAAAAAAACAATGGTTCAATGGGCCACGA
CAGACAACAAAGGTGAGTTTCGGATCATGCCCAAATCTTTAACCACAGCAGATGTTGGCAAGTGCAAGGTA
TATTTAGTGAAATCACCAAATCCAAATTGCAATGTCCCAACAAATTTCAATGGTGGAAAATCTGGTGGTTT
ATTGAAGCCTCTCCTACCACCTAAACAACCGATTACCCCTGCCGTTGTCCCTGTCCAACCACCCATGTCTG
ATTTATATGGTGTTGGACCTTTTATATTTGAAGCCTCCAGCAAAATGCCATGCGATAAGAAT**TGACCATGG**
CATGGGTACCCCGGGATCCCGTCCT
