# Supplementary material for: Structure and function of class III pistil-specific extensin-like protein in interspecific reproductive barriers
Source: BMC Plant Biol. 2019 Mar 29;19:118. doi: 10.1186/s12870-019-1728-8 (PMC6440088; doi:10.1186/s12870-019-1728-8)
Supplement: Supplementary file 7 [file 12870_2019_1728_MOESM6_ESM.docx]

Primer name	Primer sequence 5' → 3'
	**Primers to generate PIIICA gene construct**
PIIIc-aM1-F	TGCTTAGTATCAAGCTTATCGATAGATCTCCATGGCTGTTATTATTTCATCGAAGGT
PIIIc-aM2-R	CAGGTGGTGATGGCTTAGCTGGTGAAGGTG
PIIIc-aM3-F	CACCTTCACCAGCTAAGCCATCACCACCTGATCAGTCTGCAA
PIIIc-aM4-R	AGGACGGGATCCCGGGGTACCCATGCCATG
	**Primers to generate NpCt gene construct**
NpCt-F-NPIII	TGCTTAGTATCAAGCTTATCGATAGATCTCCATGGCTGTTATTATTTCATC
NpCt-R-NPIII	CGGCTTGCAGTAAACAAGGCCACGGACTGCTGGGGGCTTCCCCAAAGGTG
	**Primers to generate NtCp gene construct**
NtCp-F-NTTS	TGCTTAGTATCAAGCTTATCGATAGATCTCCATGGCAAAGGCCCTTGTTC
NtCp-R-NTTS	GGATTTACAATAAACAAGGCCACTGACGATTACAGGTTTCCTAGTAATAGGAG
	**Primers for gene construct confirmation**
	**Amplification of NtCp gene**
Sp41NTTS	TCGATAGATCTCCATGGCAAAG
CPIII7S	CATGCCATGGTCAATTCTTATC
	**Amplification of NpCt gene**
Sp41NPIII	CGATAGATCTCCATGGCTGTTA
CTTS7S	CATGCCATGGTTACTTTTTGC
	**Amplification of PIIICtrl and PIIICA genes**
Sp41-NPIII	CGATAGATCTCCATGGCTGTTA
CPIII-7S	CATGCCATGGTCAATTCTTATC
	**Amplification of RNAiPIII gene**
pdkF	GACGAAGAAGATAAAAGTTGAGAGT
7S-pdk	CCCGATACTTTCCTCGCTCA
	**Primers to detect transgenes in transformed plants**
Sp41 pdk	TGCCTCGTTCTTGAAACTGC
7S pdk	CCCGATACTTTCCTCGCTCA
	**Primers to detect DNA contamination in RNA**
PIII-F	CTAGTACTTGGCTCATTCTCAAA
PIII-R	AGAAGAGGCGATTTCTTAGC
	**Primers for qRT-PCR to check mRNA levels**
	**Amplification of Actin**
Actin-F	TGTGTTCCCTAGCATTGTGG
Actin-R	GGAGCCACACGAAGTTCATT
	**Amplification of PIIICtrl, PIIICA and NtCp**
CTD-PIII-F	TGTCCAACCACCCATGTCT
CTD-PIII-R	CCGATACTTTCCTCGCTCAC
	**Amplification of PELPIII-S**
PIII-CDS-F S/T	CCCAACAAATTTCAATGGTGG
PIII-3UTR-R-S	CCCATCTTTCTGGCTTGTATTC
	**Amplification of PELPIII-T**
PIII-CDS-F S/T	CCCAACAAATTTCAATGGTGG
PIII-3UTR-R-T	AACTCATGCTCTTATACATTATCGC
	**Amplification of NpCt**
CTD-TTS-F	CTCCCTGTTAAACTCCCCAC
7S-R	CCGATACTTTCCTCGCTCAC
