# Supplementary material for: Structure and function of class III pistil-specific extensin-like protein in interspecific reproductive barriers
Source: BMC Plant Biol. 2019 Mar 29;19:118. doi: 10.1186/s12870-019-1728-8 (PMC6440088; doi:10.1186/s12870-019-1728-8)
Supplement: Supplementary file 8 [file 12870_2019_1728_MOESM7_ESM.docx]

Amplicon 1 PIIICA (515 bp)

*TGCTTAGTATCAAGCTTATCGATAGATCTCCATGGCTGTTATTATTTCATCGAAGGT*CCTACTTATACAAC
TTTTCGTTCTAGTACTTGGCTCATTCTCAAAGCTCAGCCATGGGGAGCTGTGGCTGGAACTCCCATTGCCT
TTCGACTGGCCACCAGCGGAGATCCCATTGCCGGATATCCCATCGCCTTTCGATGGGCCTACATTCGTGCT
ACCGCCACCGTCACCATTGCCATCACCACCTCCGCCATCTCCATCACCACCTCCGCCATCACCATCACCAC
CTCCTCCGTCGACAATACCACTTATTCCTCCTTTTACCGGCGGCTTTTTGCCTCCTTTGCCCGGCTCTAAG
CTTCCTGATTTTGCCGGCCTTTTGCCTCTGATTCCCAACTTACCTGATGTGCCTCCCATAGGTGGTGGTCC
ACCTGTTAACCAGCCAAAGCCGTCGTCACCATCACCCCTAGTTAAGCCGCCACCACCAC***CACCTTCACCAG***
CTAAGCCATCACCACCTG

Amplicon 2 PIIICA (857 bp)

CACCTTCACCAGCTAAGCCATCACCACCTG*ATCAGTCTGCAA*AGCAACCACCACAACCTCCTCCAGCAAAA
CAACCATCACCTCCACCTCCTCCACCACCAGTTAAGGCACCATCTCCATCTCCAGCAAAGCAACCACCACC
TCCTCCACCGCCAGTTAAGGCACCATCTCCATCTCCAGCCACTCAGCCACCTACAAAGCAACCGCCACCGC
CGCCACGTGCTAAGAAATCGCCTCTTCTACCTCCTCCGCCACCAGTTGCTTATCCGCCAGTTATGACACCA
TCTCCATCACCGGCTGCTGAGCCACCTATTATAGCACCATTTCCATCACCACCAGCGAATCCACCCCTCAT
TCCCCGTCGACCAGCACCACCAGTAGTTAAGCCGCTTCCACCTTTGGGGAAGCCCCCAATCGTCAGTGGCC
TTGTTTATTGTAAATCCTGCAACAGCTATGGGGTTCCCACTCTGCTCAACGCCTCCCTACTCCAAGGAGCT
GTTGTGAAACTAATTTGCTACGGAAAAAAAACAATGGTTCAATGGGCCACGACAGACAACAAAGGTGAGTT
TCGGATCATGCCCAAATCTTTAACCACAGCAGATGTTGGCAAGTGCAAGGTATATTTAGTGAAATCACCAA
ATCCAAATTGCAATGTCCCAACAAATTTCAATGGTGGAAAATCTGGTGGTTTATTGAAGCCTCTCCTACCA
CCTAAACAACCGATTACCCCTGCCGTTGTCCCTGTCCAACCACCCATGTCTGATTTATATGGTGTTGGACC
TTTTATATTTGAAGCCTCCAGCAAAATGCCATGCGATAAGAATTGAC*CATGGCATGGGTACCCCGGGATCC*
CGTCCT
