# Supplementary material for: Structure and function of class III pistil-specific extensin-like protein in interspecific reproductive barriers
Source: BMC Plant Biol. 2019 Mar 29;19:118. doi: 10.1186/s12870-019-1728-8 (PMC6440088; doi:10.1186/s12870-019-1728-8)
Supplement: Supplementary file 9 [file 12870_2019_1728_MOESM8_ESM.docx]

Amplicon 1 NpCt (925 bp)
*TGCTTAGTATCAAGCTTATCGATAGATCTCCATGGCTGTTATTATTTCATC*GAAGGTCCTACTTATACAAC
TTTTCGTTCTAGTACTTGGCTCATTCTCAAAGCTCAGCCATGGGGAGCTGTGGCTGGAACTCCCATTGCCT
TTCGACTGGCCACCAGCGGAGATCCCATTGCCGGATATCCCATCGCCTTTCGATGGGCCTACATTCGTGCT
ACCGCCACCGTCACCATTGCCATCACCACCTCCGCCATCTCCATCACCACCTCCGCCATCACCATCACCAC
CTCCTCCGTCGACAATACCACTTATTCCTCCTTTTACCGGCGGCTTTTTGCCTCCTTTGCCCGGCTCTAAG
CTTCCTGATTTTGCCGGCCTTTTGCCTCTGATTCCCAACTTACCTGATGTGCCTCCCATAGGTGGTGGTCC
ACCTGTTAACCAGCCAAAGCCGTCGTCACCATCACCCCTAGTTAAGCCGCCACCACCACCACCTTCACCAT
GTAAGCCATCACCACCTGATCAGTCTGCAAAGCAACCACCACAACCTCCTCCAGCAAAACAACCATCACCT
CCACCTCCTCCACCACCAGTTAAGGCACCATCTCCATCTCCAGCAAAGCAACCACCACCTCCTCCACCGCC
AGTTAAGGCACCATCTCCATCTCCAGCCACTCAGCCACCTACAAAGCAACCGCCACCGCCGCCACGTGCTA
AGAAATCGCCTCTTCTACCTCCTCCGCCACCAGTTGCTTATCCGCCAGTTATGACACCATCTCCATCACCG
GCTGCTGAGCCACCTATTATAGCACCATTTCCATCACCACCAGCGAATCCACCCCTCATTCCCCGTCGACC
AGCACCACCAGTAGTTAAGCCGCTTC*CACCTTTGGGGAAGCCCCCA****GCAGTCCGTGGCCTTGTTTACTGCA***
AG

Gene block 1 NpCt (442 bp)
GCAGTCCGTGGCCTTGTTTACTGCAAGCCGTGCAAGTTTAGAGGGGTTAAAACTCTAAACCAAGCTTCCCC
ACTCCTGGGAGCGGTAGTGAAGCTAGTATGCAACAACACAAAGAAGACATTAGTGGAACAGGGCAAGACAG
ACAAGAATGGCTTCTTCTGGATCATGCCCAAATTCTTGTCCTCAGCAGCTTACCACAAATGCAAGGTGTTC
TTGGTCTCATCAAACAATACTTACTGTGATGTCCCAACAGATTACAATGGTGGAAAATCTGGTGCTTTGTT
GAAATACACCCCACTTCCTAAGCCACCAGCAGCTACTTCTCTCCCTGTTAAACTCCCCACATTTGATGTCT
TCACTGTTGGACCTTTTGGTTTCGAACCCTCAAAGAAGGTGCCATGCAAAAAGTAACCATGGCATGGGTAC
CCCGGGATCCCGTCCT
