# Supplementary material for: Structure and function of class III pistil-specific extensin-like protein in interspecific reproductive barriers
Source: BMC Plant Biol. 2019 Mar 29;19:118. doi: 10.1186/s12870-019-1728-8 (PMC6440088; doi:10.1186/s12870-019-1728-8)
Supplement: Supplementary file 10 [file 12870_2019_1728_MOESM9_ESM.docx]

**Gene block 1 TTS-S (273 bp)**
TGCTTAGTATCAAGCTTATCGATAGATCTCCATGGCAAAGGCCCTTGTTCTTTTTCAGCTTTCAGTTTTAT
TACTTAGCTCATTCACAGTAGTTCTTAGCCAGGAGGAAGGCATTGGGGGTTGGTTTACCACCAAACATCAT
GACCACCTTTCACCAGCTCAAGCTCCTAAGCCTCACAAAGGCAACCACCACCCCAAACATTCCCCAGCCCC
TTCACCAACTAAGCCTCCCACTTATAGCCC**ATCGAAACCACCAGTTAAACCACCGGTTAA**

**Gene block 2 TTS-S (593 bp)**
**ATCGAAACCACCAGTTAAACCACCGGTTAA**ACCACCAACTAAGGCTCCCACTTATAGCCCATCAAAACCAC
CAGCTAAGCCACCAGTTAAACCACCAACACCAACACCATCACCTTATCCTGCTCCTGCTCCTATTACTAGG
AAACCTGTAGCAGTCCGTGGCCTTGTTTACTGCAAGCCGTGCAAGTTTAGAGGGGTTAAAACTCTAAACCA
AGCTTCCCCACTCCTGGGAGCGGTAGTGAAGCTAGTATGCAACAACACAAAGAAGACATTAGTGGAACAGG
GCAAGACAGACAAGAATGGCTTCTTCTGGATCATGCCCAAATTCTTGTCCTCAGCAGCTTACCACAAATGC
AAGGTGTTCTTGGTCTCATCAAACAATACTTACTGTGATGTCCCAACAGATTACAATGGTGGAAAATCTGG
TGCTTTGTTGAAATACACCCCACTTCCTAAGCCACCAGCAGCTACTTCTCTCCCTGTTAAACTCCCCACAT
TTGATGTCTTCACTGTTGGACCTTTTGGTTTCGAACCCTCAAAGAAGGTGCCATGCAAAAAGTAACCATGG
CATGGGTACCCCGGGATCCCGTCCT
